# Computational study of NMDA conductance and cortical oscillations in schizophrenia

**DOI:** 10.3389/fncom.2014.00133

**Published:** 2014-10-17

**Authors:** Kübra Komek Kirli, G. B. Ermentrout, Raymond Y. Cho

**Affiliations:** ^1^Program in Neural Computation, Carnegie Mellon UniversityPittsburgh, PA, USA; ^2^Center for the Neural Basis of CognitionPittsburgh, PA, USA; ^3^Department of Mathematics, University of PittsburghPittsburgh, PA, USA; ^4^Department of Psychiatry, University of PittsburghPittsburgh, PA, USA; ^5^Department of Psychiatry, University of Texas Health Science Center at HoustonHouston, TX, USA

**Keywords:** NMDA receptor, schizophrenia, cortical oscillations, synchrony, computational modeling, gamma band

## Abstract

N-methyl-D-aspartate (NMDA) receptor hypofunction has been implicated in the pathophysiology of schizophrenia. The illness is also characterized by gamma oscillatory disturbances, which can be evaluated with precise frequency specificity employing auditory cortical entrainment paradigms. This computational study investigates how synaptic NMDA hypofunction may give rise to network level oscillatory deficits as indexed by entrainment paradigms. We developed a computational model of a local cortical circuit with pyramidal cells and fast-spiking interneurons (FSI), incorporating NMDA, α-amino-3-hydroxy-5-methyl-4-isoxazolepropionic (AMPA), and γ-aminobutyric acid (GABA) synaptic kinetics. We evaluated the effects of varying NMDA conductance on FSIs and pyramidal cells, as well as AMPA to NMDA ratio. We also examined the differential effects across a broad range of entrainment frequencies as a function of NMDA conductance. Varying NMDA conductance onto FSIs revealed an inverted-U relation with network gamma whereas NMDA conductance onto the pyramidal cells had a more monotonic relationship. Varying NMDA vs. AMPA conductance onto FSIs demonstrated the necessity of AMPA in the generation of gamma while NMDA receptors had a modulatory role. Finally, reducing NMDA conductance onto FSI and varying the stimulus input frequency reproduced the specific reductions in gamma range (~40 Hz) as observed in schizophrenia studies. Our computational study showed that reductions in NMDA conductance onto FSIs can reproduce similar disturbances in entrainment to periodic stimuli within the gamma range as reported in schizophrenia studies. These findings provide a mechanistic account of how specific cellular level disturbances can give rise to circuitry level pathophysiologic disturbance in schizophrenia.

## Introduction

Cognitive dysfunction is a core feature of schizophrenia that is the best predictor of functional outcome but is poorly treated by current medications (Green, 2006). One of the prevailing pathophysiologic hypotheses for cognitive disturbance in schizophrenia is the hypofunction of glutamate NMDA receptors (Javitt, 1987). NMDA receptor antagonists [e.g., phencyclidine (PCP) and ketamine] induce symptoms closely resembling schizophrenia, including the cognitive deficits (Javitt, 1987), while exacerbating symptoms in patients (Krystal et al., 1994). NMDA receptor hypofunction, arising from decreased expression or dysregulation of NMDA receptor subunits and associated proteins has been observed in patients with schizophrenia (Hahn et al., 2006; Beneyto and Meador-Woodruff, 2008). NMDA blockade through PCP and ketamine has also been used in rodent (Moghaddam and Jackson, 2003) and nonhuman primate (Gil-da-Costa et al., 2013) models of schizophrenia. Together, these findings are consistent with a role for NMDA receptor hypofunction in the pathophysiology of schizophrenia.

However, precisely how NMDA hypofunction may give rise to the physiologic disturbances is unclear. The impact on cortical gamma (30–80 Hz) oscillations is one possible mechanism receiving increasing attention. Gamma oscillations are closely associated with various cognitive and sensory processes such as working memory (Tallon-Baudry et al., 1999), attentional selection (Fries et al., 2001), and cognitive control (Cho et al., 2006). In schizophrenia, gamma oscillatory disturbances are reported in association with impaired behavioral performance across various domains including cognitive control (Cho et al., 2006), working memory (Basar-Eroglu et al., 2007; Haenschel et al., 2009), and auditory steady-state response (ASSR) (Kwon et al., 1999; Vierling-Claassen et al., 2008; Krishnan et al., 2009; Kömek et al., 2012). These observations point to cortical gamma oscillations being a relevant index of cortical activity and a possible neurophysiological biomarker (Javitt et al., 2008).

The ASSR, EEG entrainment to a repetitive auditory stimulus, has been a useful frequency-specific probe of auditory cortical functional integrity, consistently showing gamma frequency-specific reductions in schizophrenia (Kwon et al., 1999; Vierling-Claassen et al., 2008; Krishnan et al., 2009; Kömek et al., 2012). Building on reports of ASSR disturbances in response to 40 Hz but not 30 or 20 Hz stimuli, Krishnan et al. (2009) conducted a more fine-grained investigation of frequency-specificity, probing by increments of 5 Hz, finding ASSR reductions were limited to gamma-frequency (40–45 Hz) inputs. It has been postulated that these deficits could arise from alterations in fast GABAergic inhibition, which plays an important role in gamma synchrony (Bartos et al., 2007; Vierling-Claassen et al., 2008; Kömek et al., 2012), and there is strong evidence for specific disturbances in FSIs in the illness (Lewis et al., 2005). However, there is need for a more mechanistic account of how GABAergic disturbances may produce such frequency-specific ASSR findings in schizophrenia.

In the current study, we investigated how parametric variations in NMDA conductance modulate gamma oscillations in a simulated network. Rotaru et al. (2011) showed that excess NMDA conductance onto FSIs increased inhibitory spikes that disrupted the gamma rhythmic firing of pyramidal cells. In contrast, Compte et al. (2000) examined sustained gamma oscillations in a working memory model showing that decreases in NMDA conductance led to disruption of such persistent gamma activity. Together, these two studies suggest a non-monotonic modulation of network activity by NMDA receptor activity. Thus, NMDA hypofunction in schizophrenia could give rise to the observed gamma disturbances while overstimulation may also be physiologically detrimental. Such a non-monotonic relationship could also explain the region-specific differences in the effects of NMDAR antagonists on gamma oscillations (Roopun et al., 2008). To obtain a more precise picture of the relative contributions of NMDA-mediated transmission both in terms of receptor and cell type, we examined variations in NMDA vs. AMPA conductance as well as evaluating the differential role of NMDA receptors on pyramidal cells vs. interneurons. Our results showed a non-monotonic (inverted U) modulation of the network gamma power by NMDA conductance on FSIs, whereas varying NMDA conductance on the pyramidal cells showed a more monotonic modulation. Finally, following Krishnan et al. (2009) we modeled entrainment over a broad range of stimulus frequencies, finding that lower levels of NMDA conductance onto FSIs could reproduce the gamma-specific (~40 Hz) deficits observed in schizophrenia.

## Materials and methods

**Figure 1:**
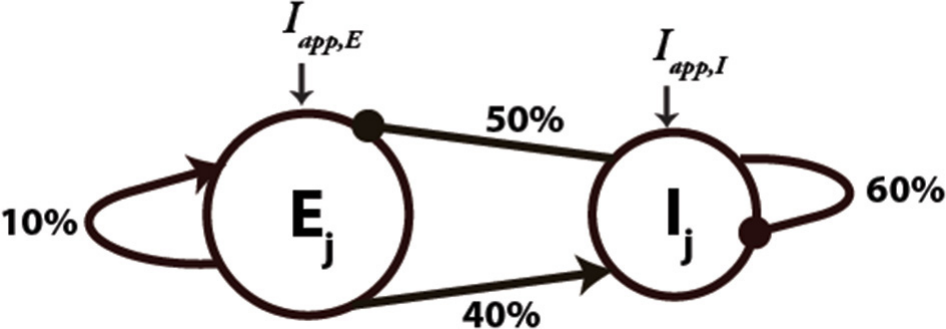
**Schematic diagram of the network architecture used for the network simulations**. E_j_ and I_j_ denote the excitatory and inhibitory neurons, respectively. An arrow denotes the connection is excitatory and a filled circle denotes that it is inhibitory. Connectivity between the neurons is random with the percentages shown on each type of connection. *I_app, E_* and *I_app, I_* denote the external applied current for the E- and I-cells, respectively.

We implemented a cortical circuit model based on prior published work (Rotaru et al., 2011), with biologically realistic network architecture and connectivity (schematic in Figure 1). The model consisted of 200 pyramidal cells (excitatory [E]) and 50 FSIs (inhibitory [I]), with biophysically realistic membrane kinetics and connection probabilities. Values for connection probabilities illustrated in Figure 1 are based upon the pyramidal and FSI data of Gibson et al. (1999). In the current network, pyramidal cells receive input from 10% of the pyramidal cells and 50% of the FSIs. In addition, FSIs receive input from 60% of the other FSIs and 40% of the pyramidal cells. Excitatory synaptic transmission was mediated by fast AMPA and slow NMDA receptors, and inhibitory synapses by fast GABA-A receptors. Both E- and I-cells are described by quadratic integrate-and-fire neuron formulation.

Membrane voltages for the E- and I-cells used in the model are governed by the following dynamics (Izhikevich, 2004):

(1) $$C_{I}\frac{dV_{I}}{dt}=I_{app,I}+g_{L,I}\frac{\left(V-V_{L,I})(V-V_{T,I}\right)}{\left(V_{T}-V_{L,I}\right)}-I_{syn}+\sigma_{I}N$$

(2) $$\begin{array}{l} C_{E}\frac{dV_{E}}{dt}=I_{app,E}+g_{L,E}\frac{\left(V-V_{L,E})(V-V_{T,E}\right)}{\left(V_{T}-V_{L,E}\right)}\\ \;-z(V-V_{K})-I_{syn}+\sigma_{E}N \end{array}$$

(3) $$\frac{dz}{dt}=-az$$

where N is white noise with coefficients σ_*I*_, σ_*E*_ for I- and E-cells, respectively, and z denotes spike-frequency adaptation with a coefficient of a (*a* = 0.01) added to the excitatory neurons to account for their observed slower firing rates (McCormick et al., 1985), *V_K_* represents the potential for the hyperpolarizing, adapting current. Every time V hits the threshold to spike, *V_T_*, *z* is incremented by d (*d* = 0.2) and V is reset to *V_R_*. Other parameters are presented in Table 1.

**Table 1:** **Model Parameters**.

Applied current to E cells (*I_app,E_*)	4 μA/cm^2^(tonic), 2.4 (periodic)
Applied current to I cells (*I_app,I_*)	0 μA/cm^2^(tonic), 0.1 (periodic)
E-cell threshold potential (*V_T,E_*)	−45 mV
I-cell threshold potential (*V_T,I_*)	−30 mV
E- and I-cell reset potential (V_R,E_, V_R,I_)	−52 mV
Adapting current potential (V_K_)	−75 mV
NMDA, AMPA equilibrium potential (V_ex_)	0 mV
GABA equilibrium potential (V_in_)	−70 mV
E and I cell leak equilibrium potential (V_L,E_, V_L,I_)	−65 mV
E and I cell membrane capacitance (C_E_, C_I_)	1 μF/cm^2^
Leak maximum conductance to E cells (g_l,E_)	0.05 mS/cm^2^
Leak maximum conductance to I cells (g_l,I_)	0.5 mS/cm^2^
Noise coefficient for Ecells (σ_*E*_)	1.0
Noise coefficient for I-cells (σ_*I*_)	0.8
NMDA decay time constant (τ_*n*_)	80 ms
AMPA decay time constant at E 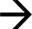 I synapses (τ_*ei*_)	1 ms
AMPA decay time constant at E 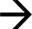 E synapses (τ_*e*_)	3 ms
GABA decay time constant (τ_*i*_)	2 ms

The total synaptic currents are given by:

(4) $$I_{syn}=I_{AMPA}+I_{NMDA}+I_{GABA}$$

where

(5) $$I_{AMPA}=g_{AMPA}s_{e}(V-V_{ex})$$

(6) $$I_{NMDA}=\frac{g_{NMDA}s_{n}(V-V_{ex})}{1+\left[Mg^{2+}\right]exp(-0.062V/3.57)}$$

(7) $$I_{GABA}=g_{GABA}s_{i}(V-V_{in})$$

In this model, NMDA currents are modeled according to the descriptions of Wang (1999) but also are also voltage-dependent as controlled by the concentration of extracellular magnesium following the formulation of Jahr and Stevens (1990) with [Mg^2+^] = 1 mM.

Every neuron to which a given cell is connected contributes its own synaptic current. Synaptic gating variables satisfy the following dynamics:

(8) $$\frac{ds_{e}}{dt}=-\frac{s_{e}}{\tau_{e}}$$

(9) $$\frac{ds_{n}}{d}t=a_{n}s_{e}(1-s_{n})-{\frac{s_{n}}{\tau }}_{n}$$

(10) $$\frac{ds_{i}}{d}t=-\frac{s_{i}}{\tau_{i}}$$

where *s_n_* is an NMDA synapse, *s_e_* is an AMPA synapse, and *s_i_* is a GABA synapse. Synaptic conductance weights are listed in Table 2. Each time an excitatory cell fires, *s_e_* is incremented by 1, and each time an inhibitory neuron fires, its corresponding *s_i_* is incremented by 1.

**Table 2:** **Synaptic conductance weights**.

NMDA onto the E-cell (g_NE_)	0.008
NMDA onto the I-cell (g_NI_)	0.008
AMPA onto the E-cell (g_EE_)	0.1
AMPA onto the I-cell (g_EI_)	0.08
GABA onto the E-cell (g_IE_)	0.25
GABA onto the I-cell (g_II_)	0.1

In the periodic drive model, both the excitatory and inhibitory cells receive additional periodic currents governed by *I_e_*(*t*) = *A_e_z*(*t*) and *I_i_*(*t*) = *A_i_z*(*t*) with *A_e_* = 70 and *A_i_* = 15 representing the amplitude of the periodic drive given to the excitatory and inhibitory neurons, respectively. Even in the periodic input case, there is a small tonic component of the applied current to account for lower baseline activity. Also *z(t)* is a transfer function defined by

(11) $$\frac{dz}{dt}=\frac{-z+P(t)}{\tau }$$

where *P(t)* is a periodic square pulse of duration 1 ms and decay time constant τ = 10 ms.

Simulations were performed in XPPAUT 6.10 (Ermentrout, 2002) using Euler's method with stepsize = 0.05 msec. The network was simulated for 10 s and divided into 1-s time bins. Local field potential (LFP) was calculated as mean voltage across E-cells. Power spectra were derived from LFPs in each time bin (Fast Fourier Transform; MATLAB, The MathWorks, Inc.). We first examined network oscillations under tonic input conditions. After applying the FFT, we identified the frequency corresponding to the peak amplitude in the power spectrum for each time bin, with amplitudes averaged over ±3 Hz windows to derive stable estimates of the peak frequency. We also examined network oscillations under periodic forcing inputs to simulate ASSR studies. Power at the driving frequency was derived as an index of network's ability to entrain across specific frequencies, ranging from 5 to 50 Hz in increments of 5 Hz. The same process was repeated for each time bin with averages calculated across the 10 different 1-s time bins.

## Results

### NMDA receptors on the fast-spiking interneurons

**Figure 2:**
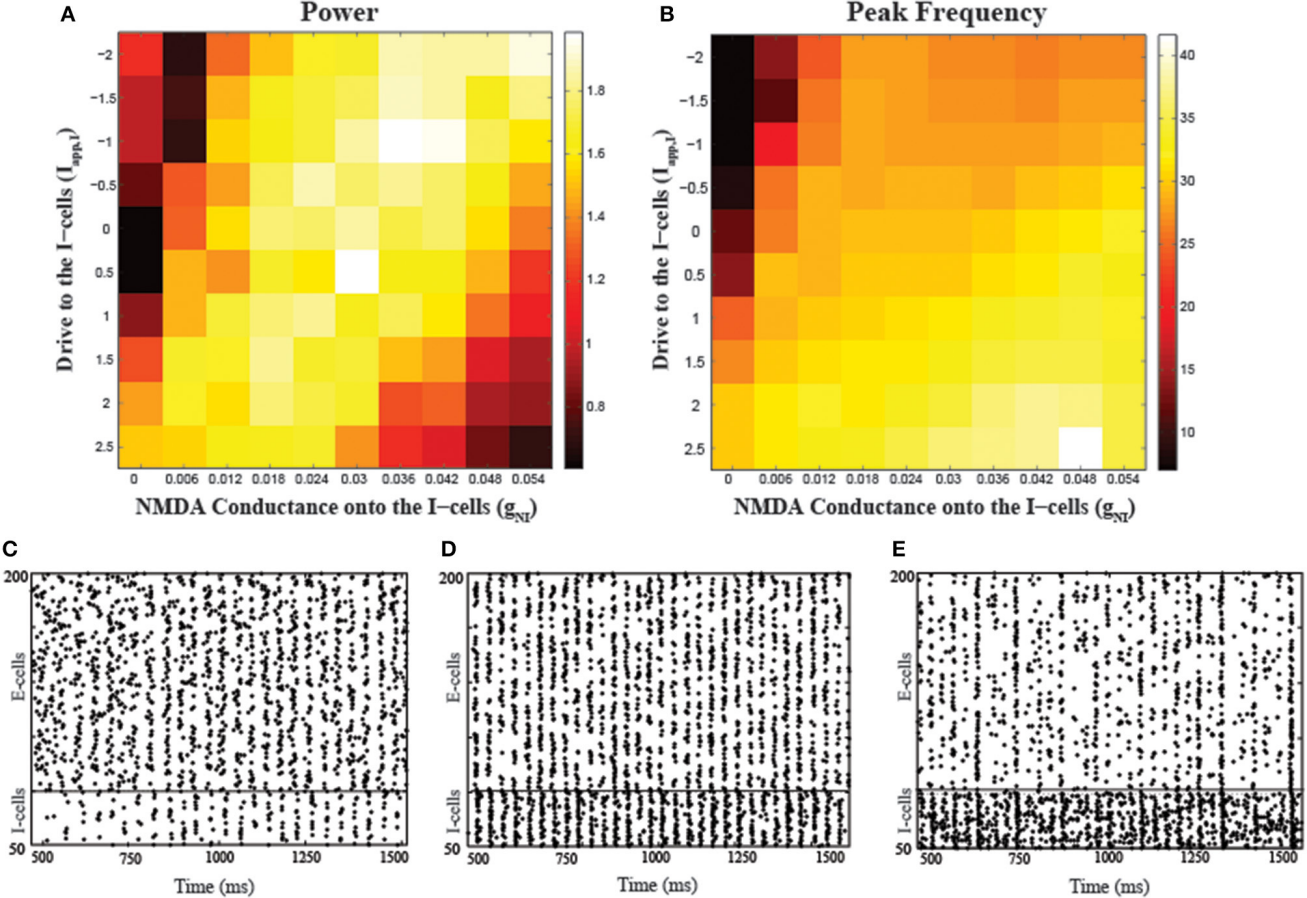
**Effects of varying the NMDA conductance (g_NI_) and the applied drive to the I-cells (*I_app, I_*) on network synchronization. (A)** The dependence of peak power on the two parameters and **(B)** peak frequency (in Hz) corresponding to the power values shown in **(A)**. Raster plots showing the network behavior during a portion of the simulation period (500–1500 ms) corresponding to g_NI_ = 0.006 **(C)**, g_NI_ = 0.020 **(D)**, and g_NI_ = 0.054 **(E)** with *I_app, I_* = 0.5 in all the conditions.

To examine the effects of NMDA receptors in FSIs on the network rhythms, we simulated a cortical model composed of quadratic integrate-and-fire neurons, examining how network gamma power varied with respect to NMDA conductance (g_NI_) changes. Figure 2 shows the results for the peak power and corresponding frequency obtained from simulations with two parameter variations, applied current to the I-cells (*I_app,I_*) and g_NI_. In addition to parametrically varying g_NI_, we also varied *I_app,I_* to examine how the modulation of network gamma by NMDA conductance on the I-cells was affected by the tonic excitatory status of the circuit. Across a range of external drive levels, gamma power had an inverted-U shaped relationship with respect to variations in NMDA conductance while peak frequency remained within the gamma range. For example, for a fixed *I_app,I_* = 0, low values of g_NI_ (g_NI_ = 0, 0.006) led to low gamma power, which increases with increases in g_NI_ (g_NI_ = 0.012, 0.018, 0.024). However, further increases in g_NI_ (g_NI_ = 0.054) caused a drop in the gamma power while the peak frequency of oscillations for this range of g_NI_ values increased slightly within the gamma range. Also, it should be noted that network gamma power displayed similar non-monotonic modulations by *I_app,I_*. At lower levels of g_NI_, there was low gamma power at both low and high values of *I_app,I_*, with high power at the mid-range optimal values of *I_app,I_*. Similar inverted-U shaped modulations of gamma power by both g_NI_ and *I_app,I_* highlight the significant role of interneuron excitability in effecting such non-monotonicity. Raster plots corresponding to three different g_NI_ values depict how firing patterns vary depending on g_NI_ (Figures 2C–E). Low levels of g_NI_ fail to provide the necessary drive to excite the I-cells and consequently, E-cells do not receive the necessary inhibition to synchronize (Figure 2C). Increasing g_NI_ leads to optimum excitation of the I-cells, providing the necessary inhibition to synchronize the E-cells (Figure 2D). However, further increases in g_NI_ lead to excessive I-cell activity, suppressing E-cell activity and resulting in low gamma power (Figure 2E).

### NMDA receptors on the pyramidal cells

**Figure 3:**
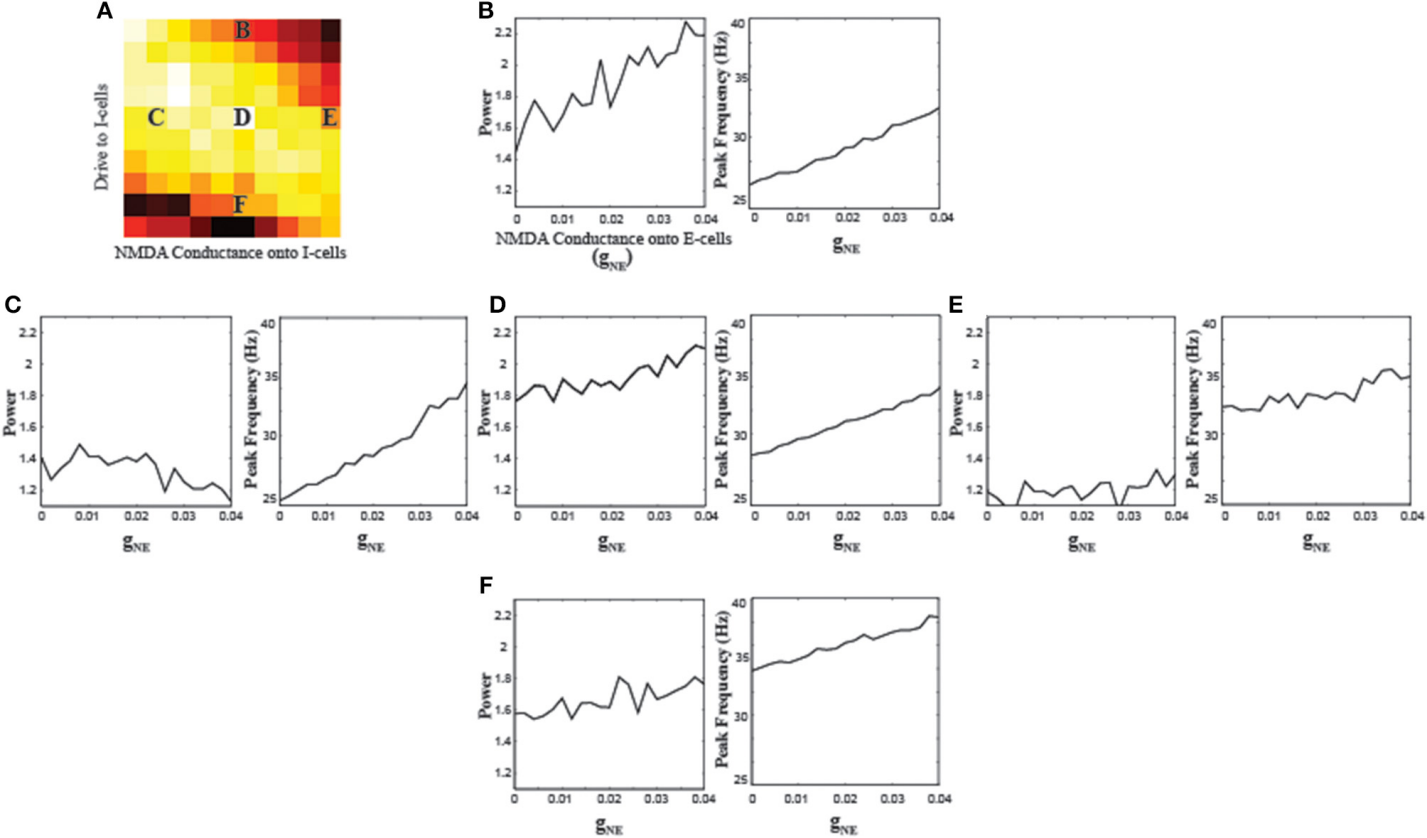
**Effects of varying the NMDA conductance onto the E-cells (g_NE_) on network synchronization. (A)** Schematic of where the parameter combinations g_NI_ and *I_app,I_* used for the simulations of g_NE_ lie. Results of varying gNE when g_NI_ = 0.03 and *I_app,I_* = −2 **(B)** g_NI_ = 0.006 and *I_app,I_* = 0 **(C)** g_NI_ = 0.03 and *I_app,I_* = 0 **(D)** g_NI_ = 0.054 and *I_app,I_* = −0 **(E)** and g_NI_ = 0.03 and *I_app,I_* = 2 **(F)**.

In order to understand the differential role of NMDA receptors on different neuron types, we also parametrically varied the strength of NMDA conductance onto the E-cells (g_NE_). Since the effects of g_NI_ depend on the overall excitability levels of the I-cells (Figure 2), we used five different g_NI_ and *I_app,I_* combinations to investigate the effects of g_NE_ (Figure 3). A consistent pattern that emerged from varying g_NE_ was an increase in the frequency of oscillations across the different parameter combinations. Along with increases in the peak frequency, the power at those frequencies also increased in most parameter combinations. The power for parameter combination “E” appears lower and more stable compared to the other combinations, consistent with the very high interneuron excitation resulting from high g_NI_ and *I_app,I_* values, suppressing the E-cells sufficiently such that increasing NMDA conductance to the E-cells cannot suffice to increase gamma power.

### NMDA vs. AMPA receptors on fast-spiking interneurons

**Figure 4:**
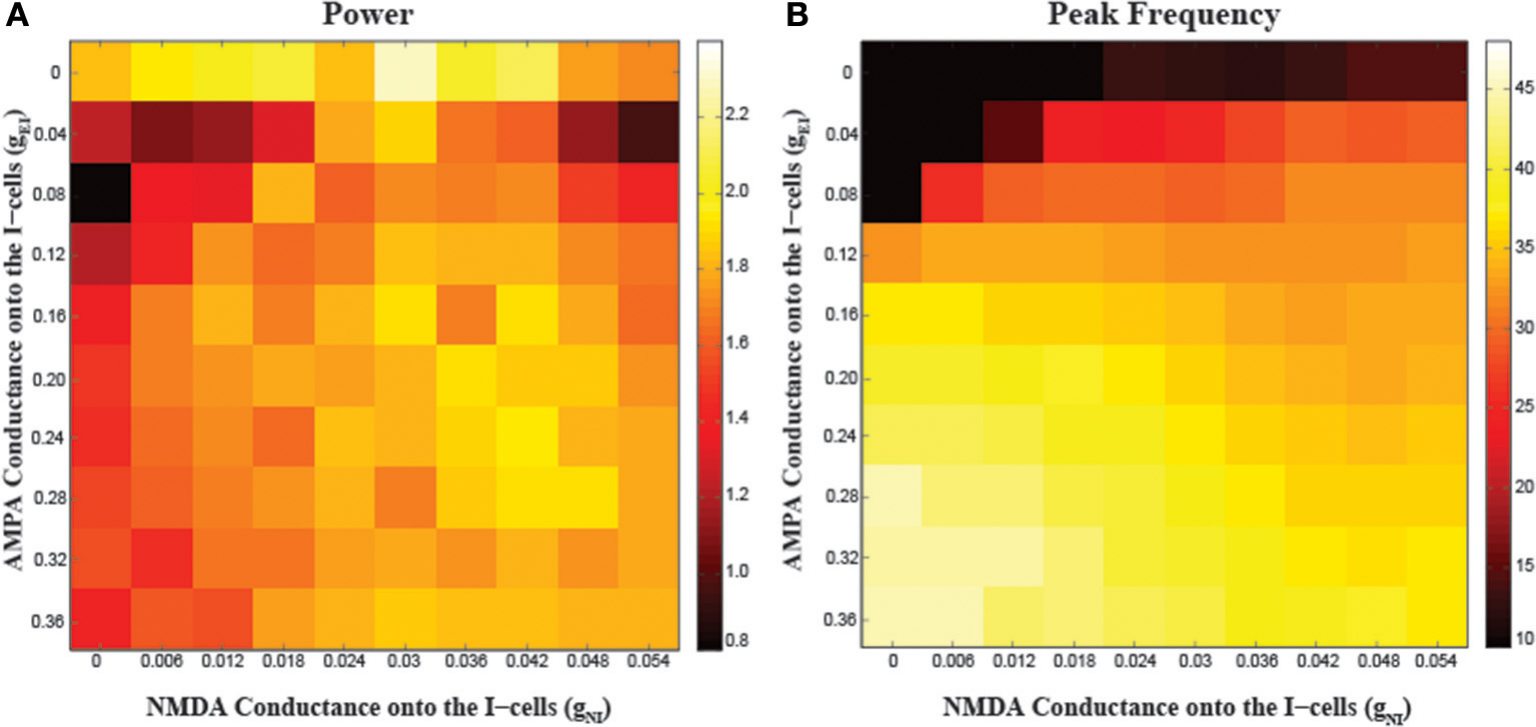
**Effects of varying the NMDA conductance (g_NI_) and AMPA conductance (g_EI_) onto the I-cells on network synchronization. (A)** The power and **(B)** peak frequency of oscillations arising from different parameter combinations.

The relative contributions of NMDA and AMPA receptors localized to the I-cells on the network oscillations are shown in Figure 4. One observation from these simulations is the importance of AMPA receptors on the generation of gamma oscillations. When AMPA conductance onto the I-cells (g_EI_) is 0, the network cannot generate gamma oscillations; the frequency of oscillations is around 10 Hz, due to the spike-frequency adaptation in the E-cells. In this regime, increasing NMDA conductance onto the I-cells does not change the frequency of oscillations. Only with increases in AMPA conductance, do gamma range oscillations emerge. In the presence of higher AMPA conductance onto the I-cells, NMDA conductance modulates gamma oscillations in a similar non-monotonic fashion as in Figure 2. For instance, for g_EI_ values of 0.04–0.12, there is an inverted-U shape relationship between network gamma power and NMDA conductance, highlighting the dependency of NMDA modulation on such critical factors as AMPA conductance and overall network excitation. Finally, with further increases in AMPA conductance, the network had significantly faster rhythms but with less power, where variations in NMDA conductance had minimal effect. These observations indicate that fast kinetics of AMPA receptors are necessary to generate and support gamma oscillations, with excessive levels having a deleterious effect. Excessive I-cell excitation by increases in either AMPA or NMDA conductance similarly suppresses E-cell activity.

### Varying periodic input frequency

**Figure 5:**
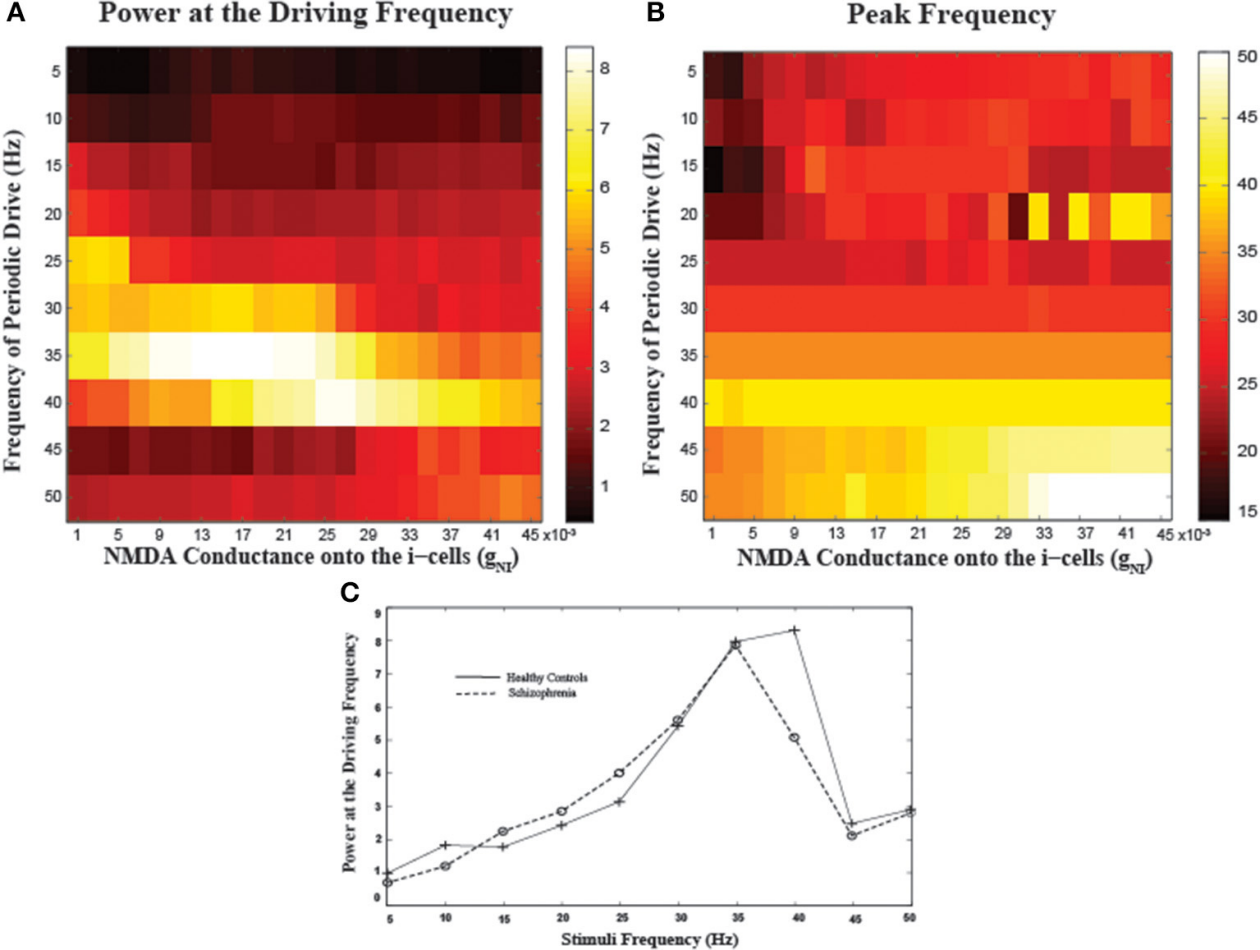
**Network entrainment to periodic stimuli as a function of NMDA conductance onto the I-cells (g_NI_). (A)** The power of oscillations at the driving frequency and **(B)** the peak frequency of oscillations emerging in the network as a function of a range of g_NI_ values. **(C)** Spectral plots for representative values of g_NI_ corresponding to healthy controls (g_NI_ = 0.025) and patients with schizophrenia (g_NI_ = 0.007). Note marked reductions in spectral power for patients specifically at 40 Hz.

To examine the frequency-specificity of the effect of NMDA conductance onto I-cells, we varied the periodic input frequencies from 5 to 50 Hz in increments of 5 Hz, following Krishnan et al. (2009) (Figure 5). Examining entrainment to 40 Hz inputs as a function of g_NI_, there is another inverted-U shaped modulation where both reductions and increases in NMDA conductance result in reduced power, similar to the tonic input case (Figure 5A). To evaluate the effects of low NMDA conductance levels as may be present in schizophrenia, we compared a mid-range value of g_NI_ = 0.025 (“healthy controls”) to a lower g_NI_ = 0.007 (“schizophrenia”) as representative of hypofunction (Figure 5C). In line with empirical findings (c.f. mean power plots in Figures 3, 4 in Krishnan et al., 2009), we observed the largest power in driving frequencies around 40 Hz, with neither group entraining well to frequencies lower than 25 Hz or higher than 45 Hz. Note the observations noted here elicited by perturbing NMDA conductance appear specific to I-cells as similar specific as similar manipulations for the pyramidal cells appear to have minimal effect (see Supplementary Figure).

## Discussion

In the current study, we investigated the role of NMDA receptors on cortical oscillations in a simulated neural network and can draw several conclusions from our observations. First, the role of NMDA receptors on the network oscillations depends on the cellular subtype that is targeted. Our results suggest that NMDA receptors localized to the interneurons have non-monotonic interactions with network gamma. This occurs through the modulation of interneuron excitability which was previously shown to be a potential mechanism underlying such inverted-U interactions with network gamma (Kömek et al., 2012). In contrast, NMDA receptors on pyramidal cells modulate cortical oscillations in a more monotonic fashion by increasing their excitability. Second, our analysis of the relative contribution of FSI NMDA and AMPA receptors on network synchrony suggest complementary roles for the different glutamatergic receptor subtypes: while some minimal AMPA conductance is necessary for the generation of gamma oscillations, NMDA receptors modulate these oscillations by altering the excitability of both the inhibitory and pyramidal cells. Finally, investigating periodic driving of the network with a broad range of frequencies following Krishnan et al. (2009) showed that NMDA hypofunction was sufficient to reproduce the pattern of their empirical observations with gamma range-specific entrainment deficits in schizophrenia.

Studies of NMDA dysfunction in schizophrenia suggest that the deficit affects FSIs more severely than the pyramidal cells. Postmortem studies show disease-specific evidence of reductions in NMDA NR2 subunit mRNA in GABA interneurons in schizophrenia (Woo et al., 2008). The functional impact may be further compounded by the observation that inhibitory local circuitry is 10-fold more sensitive to NMDAR antagonists than pyramidal neurons (Grunze et al., 1996). This is complemented by our findings that even comparable NMDAR perturbations across the cell types have a much greater functional impact for network gamma power when perturbing NMDA conductance in FSIs compared to pyramidal cells (see Supplementary Figure). Disturbances in NMDA-mediated glutamate transmission onto GABA neurons may underlie core cognitive impairments in schizophrenia such as working memory and cognitive control which are associated with gamma oscillatory disturbances (Cho et al., 2006; Haenschel et al., 2009; Minzenberg et al., 2010; Chen et al., 2014). Consistent with this, Carlén et al. (2012) used optogenetic tools to demonstrate a significant role of NMDA receptors localized to FSIs in regulating brain rhythms and cognitive functions, showing that mutant mice lacking NMDA receptors on FSIs had disrupted gamma oscillatory activity in association with working memory and associative learning impairments. Our findings provide a mechanistic account of how NMDA hypofunction could lead to reduced network gamma synchrony in schizophrenia and places this result in the broader context of a possible inverted-U shape relationship between network gamma and NMDA conductance on FSIs.

Although NMDA receptors on FSIs have been shown to be involved in gamma oscillations, the relative contributions in comparison with AMPA receptors are not clear. Fuchs et al. (2007) showed that reduced excitation of FSIs through loss of AMPA receptors leads to disturbances in spontaneous and induced gamma oscillations in hippocampal slices. However, similar disturbances were observed with knockout of NMDA receptors in FSIs (Carlén et al., 2012). Our results from parametric variations of both NMDA and AMPA conductances onto FSIs demonstrate the necessity of both glutamatergic receptor subtypes, with differential contributions shaped by differences in their excitatory postsynaptic current (EPSC) kinetics. NMDAR-EPSCs have slow kinetics, while AMPAR-EPSCs are much faster with relatively higher amplitudes (Gonzalez-Burgos and Lewis, 2012). Therefore, NMDAR-EPSCs provide more sustained excitatory drive to FSIs, which is especially critical in working memory (Compte et al., 2000) while the short lasting AMPAR-EPSCs provide the fast-timescale, large-amplitude excitatory input to the interneurons necessary for the generation and synchronization of gamma oscillations (Compte et al., 2000; Rotaru et al., 2011).

Periodic driving paradigms eliciting ASSRs have been useful for probing for gamma oscillatory disturbances in schizophrenia in a highly frequency specific manner (Kwon et al., 1999; Vierling-Claassen et al., 2008; Krishnan et al., 2009; Kömek et al., 2012). To perform a detailed examination of frequency-specificity of auditory entrainment deficits in schizophrenia, Krishnan et al. (2009) used stimuli with frequencies ranging from 5 to 50 Hz. Following their empirical design in a computational paradigm and modeling schizophrenia as reduced NMDA conductance onto FSIs, we reproduced their findings of strongest entrainment to periodic input at ~40 Hz, which was reduced in schizophrenia compared to healthy individuals. The computational mechanisms that could lead to gamma entrainment deficits in schizophrenia appear to converge on FSIs, which have been demonstrated to be critical to the dynamics of gamma oscillations in the hippocampus and neocortex (Bartos et al., 2007) and for which there is strong evidence of disturbance in the illness (Lewis et al., 2011). Prior computational work by Vierling-Claassen et al. (2008) modeled ASSR deficits in schizophrenia through increases in inhibition decay time to model the effects of decreases in the GABA transporter GAT-1 in schizophrenia (Lewis et al., 2005). More recently, we modeled the effects of reductions in cortical dopamine in schizophrenia as reduced FSI excitability, replicating similar entrainment deficits to gamma range stimuli in schizophrenia (Kömek et al., 2012). Our computational study of NMDA receptor dysfunction in FSIs also highlights the role of FSI excitation in modulating gamma entrainment and its disturbance in schizophrenia.

Despite the converging evidence for NMDA receptor dysfunction and oscillatory disturbances in schizophrenia, how perturbations in NMDA receptor function modulate these oscillations may be variable, including region-specific dependencies (Roopun et al., 2008). Administration of NMDA antagonist ketamine has been shown to increase gamma rhythms in frontoparietal areas (Pinault, 2008) while reducing gamma rhythms in superficial layers of medial entorhinal cortex (Cunningham et al., 2006). In the context of the ASSR, fine temporal sampling has revealed variability in the directionality of ketamine-induced amplitude modulations in 40-Hz ASSR in humans, with a brief decrease immediately following ketamine administration (first min) flipping to longer lasting (3–5 min post-administration) increases (Plourde et al., 1997). In hippocampus, ketamine has also shown differential effects depending on whether it is *in vivo* (Ma and Leung, 2007) or *in vitro* (Dickinson et al., 2003). Together, these findings appear discordant, but the variability clearly could derive from a number of factors that vary across the studies, including specific brain region, species, *in vitro* vs. *in vivo* preparations, the timing of measurement relative to drug administration, as well as drug dose. It is also important to point out that ketamine administration, while mimicking some of the features of schizophrenia, may be limited as a specific test of NMDA hypofunction in the illness as it effects a number of other neurotransmitter systems (e.g., opioid, sigma, adrenergic, cholinergic, serotonergic) and is also known to modulate glutamate and dopamine levels in a dose- and region-dependent manner (Moghaddam et al., 1997). Further, the findings of ketamine increasing ASSR gamma power (Plourde et al., 1997; Vohs et al., 2012) contrast with the consistent findings of reductions in schizophrenia. Thus, more refined pharmacologic tests of NMDA hypofunction may be needed to empirically test whether this mechanism could reproduce the findings in the illness.

Our findings from parametrically varying NMDA conductance onto FSIs suggest that the effects of perturbing NMDA function can depend critically on the baseline excitability levels of the inhibitory circuitry, accounting for some of the discrepant findings across studies. Although network gamma power generally has an inverted-U shaped dependence on NMDA conductance in FSIs, the specific shape and offset of the curve may vary with the level of network excitation, potentially contributing to the differential effects of drugs like ketamine in different contexts. Interestingly, decreases in evoked ASSR gamma power in schizophrenia have been attributed to increases in baseline gamma power (Spencer, 2012). Since determination of ASSR power typically involves subtraction of the pre-stimulus baseline power, any increases in the baseline would result numerically in a reduced ASSR magnitude. The observation of baseline gamma increases, however, were based on averages of per-trial power determinations as opposed to the typical approach in ASSR studies of deriving evoked power from already-averaged baseline EEG which has minimal amplitude due to phase cancelation across trials. Thus, while observations of increased baseline gamma power in schizophrenia certainly merit further investigation, it may have relatively little relevance to observations of reduced evoked gamma power in studies of the ASSR in schizophrenia.

Dopamine modulates cortical activity through D1 receptors in a non-monotonic, inverted-U fashion (Williams and Castner, 2006). In our prior computational work, we showed that dopamine modulation of FSI excitability produced an inverted-U shaped relationship with network gamma power (Kömek et al., 2012), modeling dopamine effects as changes in FSI excitability, based on empirical observations of dopamine's suppression of a resting leak K^+^ as well as other K^+^ currents in GABAergic interneurons (Gorelova et al., 2002). However, dopamine has diverse effects in different brain regions through varying mechanisms. Dopamine can also have differential effects on glutamate receptor-mediated responses (Cepeda and Levine, 1998), with D1 receptors potentiating NMDA receptors whereas D2 receptors attenuating primarily through AMPA receptors (Cepeda et al., 1993). Thus, dopamine's inverted-U relation with network gamma power could be mediated both by direct effects on FSI excitability and also through its modulating role on NMDA receptors which, as we have shown here, can also give rise to the inverted-U relationship with gamma oscillations.

Other modeling studies have also investigated the role of NMDA receptors in gamma oscillations. Rotaru et al. (2011) highlighted the importance of AMPA receptors in the generation of gamma oscillations, which decreased significantly with increases in NMDA conductance onto the FSIs. In the context of a spatial working memory model, Compte et al. (2000) found that sufficient NMDA conductance (onto excitatory cells) was necessary for persistence in delay-related activity as decreases in the overall NMDA conductance led to unstable persistent activity and eventual abolishment. Finally, in a modeling study examining the role of various cortical circuit abnormalities on gamma oscillations and network excitability in schizophrenia, Spencer (2009) examined the effects of reduced NMDA input to FSIs. While the focus was on the initial reductions in NMDA from the putative normal state giving rise to gamma increases, interestingly, extending this to complete reductions in NMDA-mediated input gave rise to eventual decreases in gamma. Thus, there is broad consistency across computational studies supporting an inverted-U dependence of gamma on NMDA conductance.

The interactions between dopaminergic and glutamatergic systems likely have direct clinical relevance to schizophrenia. Ketamine and PCP, which lead to schizophrenia symptomatology in healthy controls, also modulate the dopamine D2 and serotonin 5-HT2 receptors (Kapur and Seeman, 2002). Interestingly, acute administration of the antipsychotic clozapine has been shown to potentiate interactive D1 and NMDA-mediated enhancement of post-synaptic glutamate potentials (Chen and Yang, 2002), and to improve deficits in animals treated with NMDA antagonists (Malhotra et al., 1997; Rompala et al., 2013), possibly explaining some of the cognitive improvements associated with clozapine treatment. The current study, with a focus on NMDAR activity together with our prior computational work on the role of dopamine (Kömek et al., 2012) offers potential common mechanistic framework for explaining its clinical efficacy, with a convergence on ameliorating disturbances in interneuron excitability and associated cognitive impairments.

This study had a number of limitations. First, the inverted-U relationship between gamma oscillatory activity and NMDA conductance in FSIs was posited on the grounds of prior computational and empirical studies that individually showed increases or decreases in gamma power under different conditions. However, direct empirical evidence of this relationship at the within-subject level is lacking. Our simulation results, however, offer support for such a putative relationship and could serve as a strong prediction for a systematic study of the effects of broad-ranged, parametric variations in NMDA conductance on network gamma. Second, while our study primarily examined the effects of varying NMDA conductance in FSIs, future studies could investigate interactions with the level of excitatory drive to the pyramidal cells, which is also important for gamma oscillation generation, in addition to the time constant of inhibition (Kopell et al., 2010). Finally, our model only accounted for putative effects of immediate changes in NMDA conductance, while empirical studies point to dynamic changes in effects even at short time scales post-acute administration (Moghaddam et al., 1997; Plourde et al., 1997). Future work could investigate the effects of chronic hypofunction and potential adaptive mechanisms that may be critical for accurately characterizing pathophysiologic processes in schizophrenia in a neurodevelopmental context.

In summary, this study provides a computational account of the role of NMDA in gamma oscillations and how its dysfunction in schizophrenia may give rise to the resulting gamma disturbances in the illness. Although we used a cortical model, the relevance of the findings and principles could extend to other brain regions such as the hippocampus. Further work could be directed at elucidating how NMDA dysfunction and specific cognitive impairments in schizophrenia such as in working memory (Driesen et al., 2013; Rompala et al., 2013) could be mediated by gamma oscillatory disturbances. Understanding such pathophysiologic links between synaptic dysfunction and cognitive disturbance will be crucial for novel treatment development in schizophrenia.

### Conflict of interest statement

The authors declare that the research was conducted in the absence of any commercial or financial relationships that could be construed as a potential conflict of interest.
