# Supplementary material for: Computational study of NMDA conductance and cortical oscillations in schizophrenia
Source: Front Comput Neurosci. 2014 Oct 17;8:133. doi: 10.3389/fncom.2014.00133 (PMC4201161; doi:10.3389/fncom.2014.00133)
Supplement: Supplementary file 1 [file Image1.PDF]

Supplementary Material

Computational study of NMDA conductance and cortical oscillations in schizophrenia

Kübra Kömek Kırlı[1,2], Bard Ermentrout[2,3], Raymond Y. Cho[2,4,5]*

[1]Program in Neural Computation, Carnegie Mellon University, Pittsburgh, PA, USA
[2]Center for the Neural Basis of Cognition, Pittsburgh, PA, USA
[3]Department of Mathematics, University of Pittsburgh, Pittsburgh, PA, USA
[4]Department of Psychiatry, University of Pittsburgh, Pittsburgh, PA, USA
[5]Department of Psychiatry, UT Health Science Center at Houston, Houston, TX, USA

*** Correspondence: :** Raymond Y. Cho, UT Health Science Center at Houston, Department of Psychiatry and Behavioral Sciences, 1941 East Road, Rm. 3154, Houston, TX, 77054, USA.
raymond.y.cho@uth.tmc.edu

1. Supplementary Figures and Tables

1.1. Supplementary Figure 1

Our results with tonic input involving the parametric variations in NMDA conductance onto the pyramidal cells and FSIs suggested that NMDA conductance onto the FSIs was the main factor in giving rise to the inverted-U relationship, which is why only parametric variations in NMDA conductance onto the FSIs are presented in the section describing entrainment to periodic input. We also investigated the effects of reductions in NMDA conductance onto the pyramidal cells as well as the effects of various degrees of reductions in NMDA conductance onto the FSIs. Results are presented in Supplemental Figure 1 where we compare the unperturbed 'healthy control' state (gne=0.008 and gni=0.025), to four different cases involving NMDA reductions. Schizophrenia was operationalized as very low NMDA conductance onto the FSIs (gni=0.007) as in the main body the manuscript. We included three additional cases: severely reduced NMDA onto the pyramidal cells (very low gne; gne=0.002) with intact NMDA onto the FSIs; moderate reductions in NMDA onto both the FSIs and pyramidal cells (low gne, low gni; with gne=0.004 and gni=0.019), and finally moderate reduction in NMDA onto the pyramidal cells with severe reduction in NMDA onto the FSIs (gne=0.004 and gni=0.007). As illustrated in the figure, there are marked reductions in gamma power for the cases with reduced gni, either alone or in combination with gne. Reduced gne alone reproduces an entrainment profile more similar to the healthy controls than patients with schizophrenia. The changes to gne appear neither necessary nor sufficient to give rise to gamma entrainment deficits. These findings highlight the important role of NMDA conductance onto the FSIs in giving rise to schizophrenia-like entrainment deficits.

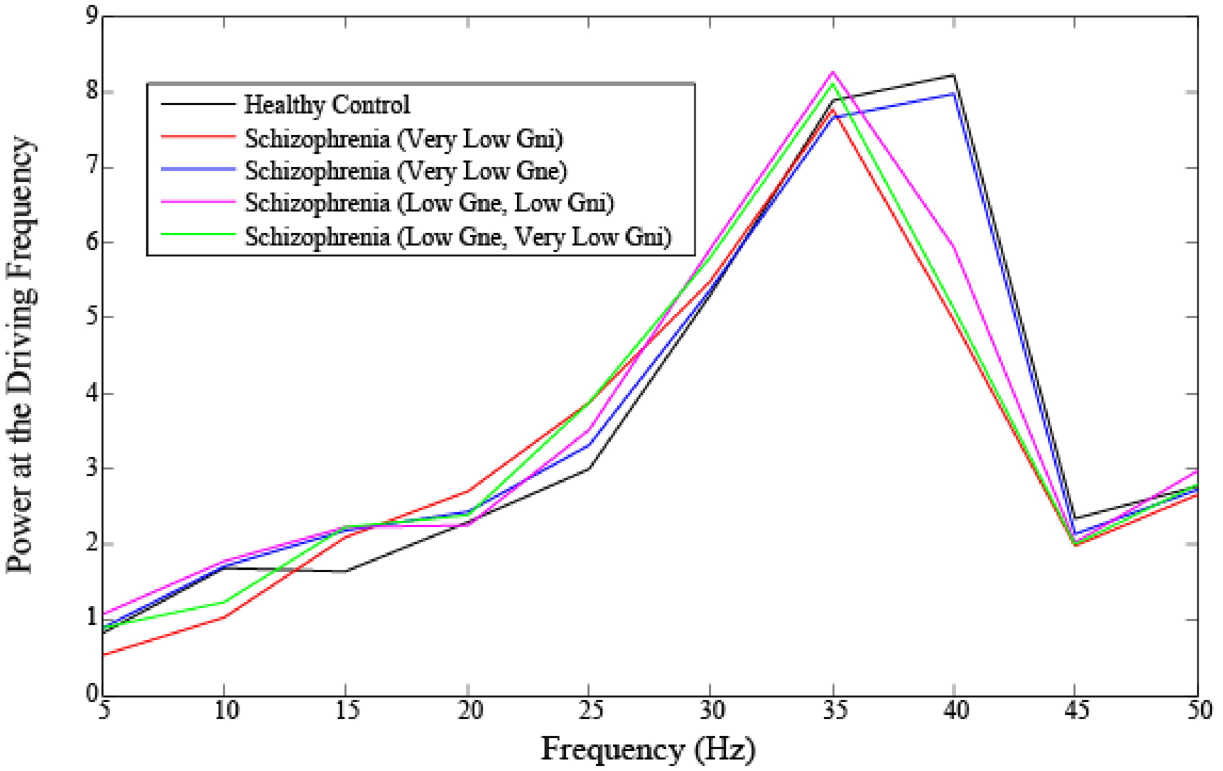

Supplementary Figure 1. Effects of various NMDA conductance reductions on network entrainment to periodic stimuli. Entrainment profile for the optimum parameters representing the healthy control state (black line) and comparison with various NMDA conductance reductions onto the pyramidal cells (g_{ne}) and the FSIs (g_{ni}). Schizophrenia is operationalized as very low g_{ni} (red line). Additional cases include severely reduced g_{ne} with intact g_{ni} (blue line), moderate reduction in both g_{ne} and g_{ni} (pink line), and finally moderate reduction in g_{ne} with severe reduction in g_{ni} (green line). Note marked reductions in spectral power for the cases with reduced g_{ni}, either alone or in combination with g_{ne}. Reduced g_{ne} alone has minimal effect.

1.2. Supplementary Figure 2

We explored whether our findings from periodic input simulations could be reproduced in a larger scale network. The network consisted of 1000 pyramidal cells and 250 FSIs with the same connection probabilities, with fine-tuning of other parameters for synaptic conductances and normalizing for large network size. Given the larger scale, simulations were implemented in the Julia programming language which greatly enhanced the computational efficiency over XPP. The findings with the larger network are similar to our original network, summarized in Supplemental Figure 2. As with the smaller network, we see differences in entrainment to periodic input between the healthy control state and schizophrenia state modeled through reductions in NMDA conductance onto the FSIs. With the larger network, differences between the healthy control and schizophrenia span a somewhat wider range of input frequencies (20-45 Hz) but with the most prominent differences in the gamma frequency range (30-40 Hz). Our results with the larger network provide further support for the potential significance of disturbances in the NMDA conductance onto the FSIs in giving rise to deficits the gamma-range specific entrainment deficits observed in schizophrenia.

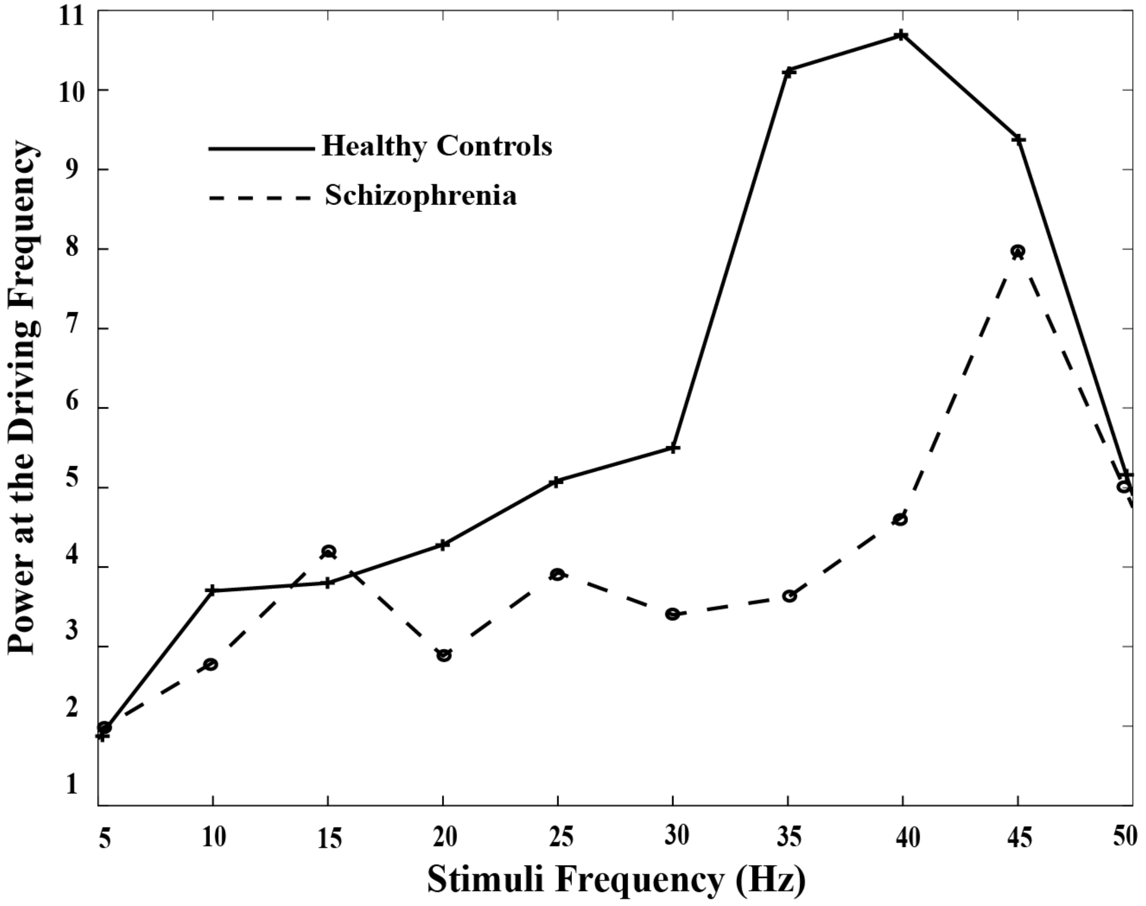

Supplementary Figure 2. Comparison of network entrainment to periodic stimuli of healthy control vs schizophrenia. Spectral plots for representative values of g_{ni} corresponding to healthy controls (optimum g_{ni}) and patients with schizophrenia (low g_{ni}).
